# An Investigation of Non-Linear Strength Characteristics of Solidified Saline Soils in Cold Regions

**DOI:** 10.3390/ma15217594

**Published:** 2022-10-28

**Authors:** Qian Ding, Zheng Hu, Shuai Huang, Kezheng Chen, Yanjie Liu, Lin Ding

**Affiliations:** 1 School of Hydraulic and Electric Power, Heilongjiang University, Harbin 150080, China; 2 School of Forestry, Northeast Forestry University, Harbin 150040, China; 3 College of Engineering and Technology, Northeast Forestry University, Harbin 150040, China; 4 School of Civil Engineering, Heilongjiang University, Harbin 150080, China

**Keywords:** solidified saline soil, cold regions, freeze–thaw cycles, damage models, Weibull distribution

## Abstract

To date, the modelling of constitutive equations of solidified frozen saline soil have seldom been studied. This paper presented the formulation of a damage constitutive model for solidified saline frozen soil considering both freeze thaw cycles (FTCs) and salinities. To model the solidified frozen saline soil, the unconfined compression strength test (UCST) and consolidated undrained (CU) triaxial shear test were conducted under three ambient temperatures (20, –10, and –20 °C), five ages (3, 7, 14, 28, and 90 d), six salinities (0, 1, 2, 3, 4, and 5%), and four FTCs (0, 5, 10, and 14 times) in this research. The UCST results showed that the unconfined compressive strength (UCS) of the solidified saline soils at an age of 14 days can reach 75% of the maximum UCS, which basically meets the engineering construction requirements. The range of the rate of strength loss as affected by salinity was 16.2% to 75.65%, while the coupling effect of salt and frozen conditions amplified the rate of strength loss. Affected by increasing salinity, the rate of strength loss of frozen soils was magnified by a factor of 1.2 to 3.7 compared to thawing soils. Likewise, the CU triaxial shear test showed that the rate of strength loss of shear strength was amplified by the coupling effect of FTCs and salt erosion. With increased FTCs, the strain threshold of Young’s modulus was gradually pushed backward, which was similar to the effect of salinity. Remarkably, the damage constitutive model performed better than conventional constitutive models for the solidified saline soil under the salt–freezing coupling effect.

## 1. Introduction

China has the third-largest frozen ground region in the world, and frozen ground covers about 56.3% of the land area in China [1,2]. Climate change has greatly affected the infrastructure in frozen ground locations, causing problems such as mud pumping-induced cracks and frost-related heaving on roads, and landslides induced by freeze–thaw [3,4,5,6]. Meanwhile, the second-largest frozen ground region and the largest carbonate-stained land area, of approximately 3.2 × 10^4^ km^2^, is located in Northeast China. Influenced by freeze–thaw cycles (FTCs) and salinization, the physical properties of the soil have suffered great deterioration [7,8]. With the economic development of society, engineering construction in cold regions is gradually receiving more and more attention from researchers, and the engineering of cold region saline soils represents a major challenge for the geotechnical engineering community. Therefore, research on the salt–thermal coupling of frozen soil is of great importance [9,10,11].

Recently, a large number of research studies have focused on the effects of FTCs on soil. Experimental testing showed that the macrophysical properties of soil were significantly influenced by FTCs. For example, the UCS and shear strength of soil deteriorated with the development of FTCs process [12]. The soil porosity and saturated hydraulic conductivity gradually increased following subjection to 30 FTCs. The microphysical properties of soil, such as the pore size, microstructure, and texture, were similarly impacted by the FTCs effects [13]. These effects of FTCs can have a superimposed effect with salt erosion and show strong non-linear physical properties [14]. Therefore, study on the mechanism of this deterioration effect is extremely important in engineering construction.

The poor physical properties of saline soil increase the likelihood of engineering geological hazards; these include melt sinking, salt heaving, and salt corrosion [15]. Therefore, engineers usually take certain foundation treatment measures (e.g., landfill replacement, solidified saline soil, and compacted soil) to ensure that the infrastructure is safe for building on saline ground [16,17,18]. In the past, researchers have carried out a large number of studies on saline soil improvement. Generally speaking, saline soil improvement methods have typically included physical improvement, chemical improvement, electro-osmosis improvement, and biological improvement [19,20,21,22,23,24,25,26,27,28,29].

Among the chemical improvement methods, the most typical and economical is the inorganic improvement method, in which lime, fly ash, and cement are added to the soil to improve its physical properties through a series of hydration reactions and the reconstitution of the soil skeleton [30,31,32]. This method is widely used in the engineering construction process [33,34,35]. Previous works have shown that the inorganic improvement method is significantly effective in dealing with the effect of saline erosion and FTCs, and markedly enhances the mechanical properties of saline soil [36,37,38,39]. Research on the physical properties of the inorganic improvement of saline soil under FTCs has a substantial prospect of engineering application.

Although previous research has shown that inorganic amendments are very economical and effective means of improving the strength of saline soils [40,41], most of the research has focused on the variation patterns of macromechanical properties and the microstructural characteristics [42,43], and few researchers have carried out research on the modelling of solidified saline soils. Traditional strength criteria and constitutive models have rarely been applied to the study of saline soil under the effect of FTCs [44,45,46,47,48,49,50]. In addition, due to the complexity of the modified saline–alkali soil as an artificially remodeled soil structure, its compaction and strength are high, and the stress damage has elastic-brittle characteristics similar to concrete, as well as the stress characteristics of a soil body [51]. Meanwhile, caused by the effect of FTCs and the effect of water salt migration, the saline soil exhibited complex physical properties, leading to irreversible damage to soils [52,53]. Thus, it is difficult to use a model to describe the mechanical properties of improved saline soils under the effect of FTCs.

In the vast frozen saline ground, engineers are desperate to find reliable physical models of modified saline soil under the effect of FTCs and to solve the engineering problem of the coupling effect of frost and salt leading to complex strength deterioration. Considering that the physical properties of the modified saline–alkali soil are between concrete and soil, this paper takes improved saline–alkali soil as the research object and draws on the concrete damage principal theory, combined with the actual mechanical properties of improved soil, to study the damage evolution law under the coupling effects of freezing and salt load conditions.

## 2. Test Materials and Methods

### 2.1. Test Materials

Test soil was taken from the Songnen Plain (126.28 °E, 46.65 °N) at a depth of 2.0~4.0 m below the existing ground surface. Based on the Standard of Test Methods of Soil for Highway Engineering (JTC3430-2020), the specific gravity of the soil was measured by density bottle methods, the liquid–plastic limit of the soil was determined by the liquid–plastic limit combined device, and the uniformity coefficient and coefficient of curvature were calculated by the size distribution test. Its main physical properties are shown in Table 1. For this study, the test soil samples were prepared manually as carbonate saline soil. The inorganic amendments used in the tests were lime, ordinary silicate cement, and primary fly ash. The lime is calcium lime powder with CaO content of 82.4%, produced by Yichun Huihui Technology Co., Ltd., Xinyu, China, the cement is P42.5 produced by YATAI Group Harbin Cement Co., Ltd., Harbin, China, and the primary fly ash selected was low calcium fly ash with a particle size of 42 μm and a burn vector of 2.02%, produced by Heilongjiang Thermal Power Company, Harbin, China. The main oxides are shown in Table 2.

### 2.2. Experimental Program

The tests were conducted using the WDW-100 uniaxial produced by Jinan ShiDaiShiJin Testing Machine Co., Ltd., Jinan, China, and GDS advanced dynamic and static permafrost triaxial test systems developed by the GDS, UK. The compressive strength test soil sample was based on a triple-ash solidified saline soil with a mass ratio of 3:4:6:87 of lime, cement, fly ash, and saline soil. The factors of temperature, age, and salinity were set to simulate a cold temperature environment. For this purpose, 180 specimens were made for UCST and 72 specimens were made for CU triaxial shear test. The diameter of the test sample was 39.1 mm and the height was 80 mm. To ensure the accuracy of our tests, the mass error of all specimens was controlled within 2 g and the height was controlled within 2 mm. The test factors and levels are shown in Table 3.

### 2.3. Experimental Methods

In road pavement sub-base construction, the unconfined compressive strength of solidified saline soils at different ages acts as an important basis for judging whether the strength requirements of the pavement sub-base are met. Therefore, this research used a microcomputer-controlled electronic universal testing machine (WDW-100E) to test the unconfined compression resistance of solidified saline soil, with the press descending at a rate of 2.0 mm/min. The microcomputer automatically collected the test data every 2 s, continuing after the peak pressure, with a 4% to 5% strain stop. The consolidated undrained test was carried out using the GDS advanced dynamic static frozen soil triaxial test system, prepared, maintained, and freeze–thaw cycled in accordance with the specifications, with the specimens being saturated by vacuum extraction followed by backpressure saturation to ensure a saturation of 90% or more before testing. The consolidation process was isotropic consolidation with a shear rate of 0.08 mm/min. The test factors and levels are shown in Table 3.

## 3. Test Results and Analysis

### 3.1. UCST Results

**Figure 1:**
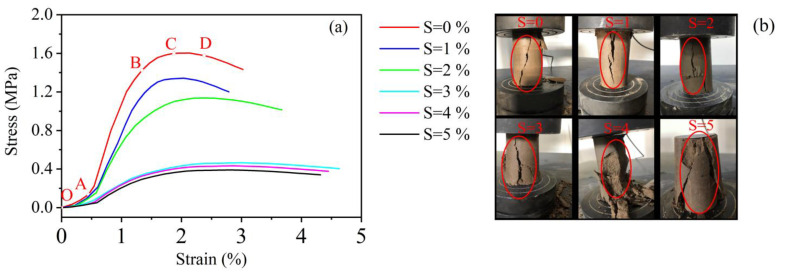
(**a**) Types of stress–strain curves (S represents salinity) and (**b**) compressive failure types for solidified saline soils with different salinities at an age of 14 days.

Figure 1 shows the stress–strain curves and damage patterns of the specimens at an age of 14 d. The stress–strain curve can be divided into five stages. Stage 1, with salinity equal to 0, for example, is the compaction stage (OA), where the pores of the solidified saline soil start to close with increasing pressure and become smaller in volume. Stage 2 is the elastic deformation stage (AB), where the stress–strain relationship is linear. After the OA stage of compaction, the original pore space is further compressed, and no cracks are produced at this stage. After further compaction, the solidified saline soil starts to produce cracks, the longitudinal volume starts to become larger, and the slope of the curve starts to become smaller, which means that the stress growth rate is smaller than the strain growth rate. After point C the slope of the curve changes less rapidly and the resulting crack quickly becomes larger and more voluminous; this is the fourth stage, known as the damage stage (CD). The fifth stage, after point D, is referred to as the post-damage stage. As the salinity rises, the compaction stage becomes longer, the elastic stage becomes shorter, the yield limit value becomes lower, and the specimen crumbles significantly in the post-damage stage.

**Figure 2:**
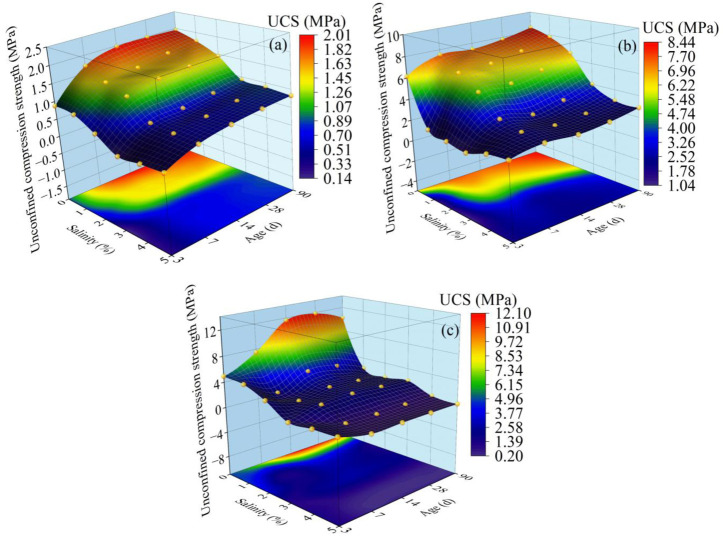
Effect of salt content and age on compressive strength of improved soils. Notes: (**a**) Ambient temperature of unconfined compression test (UCST) at 20 °C; (**b**) ambient temperature of UCST at −10 °C; (**c**) ambient temperature of UCST at −20 °C.

As shown in Figure 2, when the age is more than 7 d, the UCS of solidified saline soils decreases with increasing salinity, and the rate of strength loss *K* increases with increasing salinity. At an age of 3 d, with increasing salinity, the UCS exhibits a decreasing trend followed by an increasing trend. Thus, when the age is brief, the water in the soil does not fully participate in the hydration reaction, while the salinity is low and the salt is almost completely dissolved in the water; then, as the salinity increased, the salt produces a deteriorating effect in the soil; as the salinity exceeds the brine saturation threshold, and then as the salinity increases, the salt will precipitate in the water and become part of the soil skeleton structure, increasing the UCS. Meanwhile, when the age is lengthy, the hydration reaction occurs in its entirety, and the hydration reaction causes the soil pores to be completely filled, producing compact soil. At this time, with increasing salinity the degree of salt erosion to the soil skeleton structure gradually increases.

### 3.2. The Rate of Strength Loss

In order to analyze the effect of salinity on the UCS of solidified saline soils, the rate of strength loss due to salinity, *K*, is defined as follows:

(1) $$K=\frac{q_{0max}-q_{imax}}{q_{0max}}\times 100\%$$

where *K* is a dimensionless constant, $q_{0max}$ is the maximum UCS with a salinity of zero, and $q_{imax}$ is the maximum UCS for different salinities.

**Figure 3:**
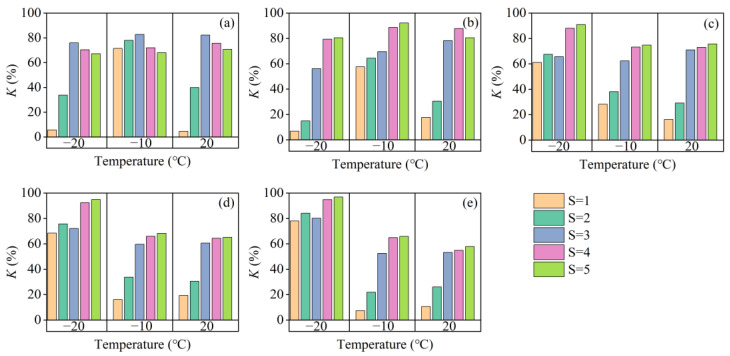
Rate of strength loss by salinity, with temperatures and ages. (**a**) Under the age of 3 days; (**b**) under the age of 7 days; (**c**) under the age of 14 days; (**d**) under the age of 28 days; (**e**) under the age of 90 days.

In Figure 3, the results of the rate of strength loss show that the rate of strength loss increased with the decreasing of temperature and the increasing of salinity when the age was more than 7 days. It is clear that the salt–freezing coupling effect will amplify the rate of strength loss. On the one hand, the lower the temperature, the more significant the frost heave effect on the soil. One the other hand, the low temperature produces lower brine saturation. Under the effect of frost heave, the soil becomes very dense; however, affected by low temperature, the crystallization of salt breaks down the dense soil structure and reduces the strength of the soil [54].

### 3.3. Age–Strength Relationships

As indicated in Figure 2, the UCS of solidified saline soils increases with age at temperatures above −20 °C and salinities below 4%. This is due to a series of hydration reactions occurring during the curing process of solidified saline soils. At this point, the soil skeleton of the lower salinity specimens consisted mainly of the hydration reaction products of the curing agent. For salinities greater than or equal to 4%, the compressive strength at an age of 3 d was greater than that at age 7 d. This is because the reaction rate of NaHCO_3_ and Ca(OH)_2_ is faster than the hydration reaction rate of the curing agent, which preferentially produces the soil skeleton [55]. However, as the age of the curing agent increases, the hydration reaction products are broken down before the soil skeleton can re-form, which causes the compressive strength curves of specimens with a salinity level of greater than 4% to first decrease and then increase. This means that when saline soils cured with inorganic amendments exceeding 4% are still cured at a strength of 3 d to 7 d according to conventional construction guidelines, this will cause significant damage to the project. In order to avoid this hazard, it is necessary to refer to the strength after 14 d of curing.

## 4. Non-Linear Strength Characterization

### 4.1. Changes in the Stress–Strain Relationship for Improved Soils for Different FTCs and Salinities

**Figure 4:**
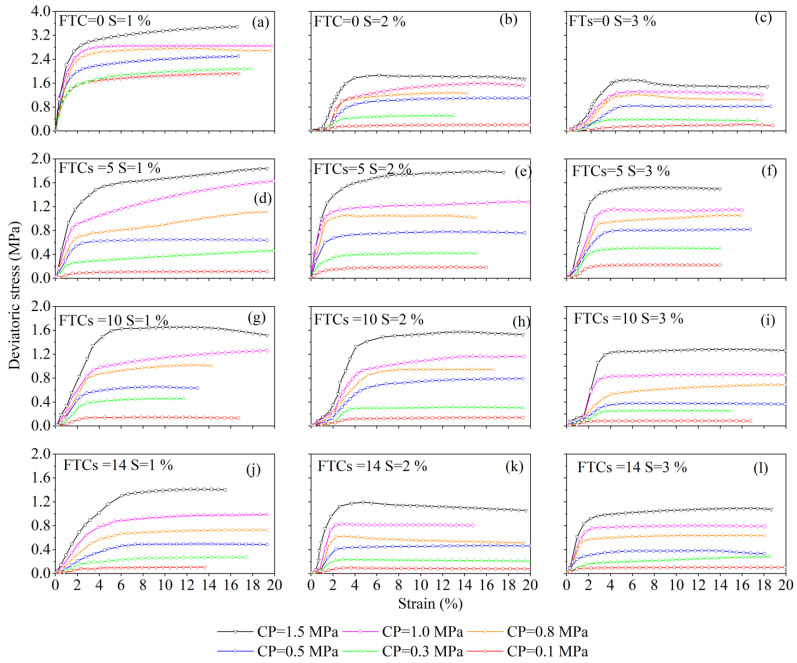
Bias stress–axial strain curves for different freeze–thaw cycles and different salinity content. Notes: FTCs represents freeze–thaw cycles, S represents salinity, and CP represents confining pressure.

The slope of the stress–strain curve for improved soils under different FTCs and different salinities is shown in Figure 4. The slope of the stress–strain curve for improved soils under the same FTC and the same salinity gradually increases with an increase in the confining pressure. The slope of the stress–strain curve decreases significantly with an increase in salinity when FTCs = 0, because the soil is improved when the salinity equals 1% and the internal structure is tight; when the salinity increases, pores develop in the solidified soil and there are fillers in the pores [56]. The slope of the stress–strain curve decreases significantly with a confining pressure (CP) ≤ 0.5 MPa, and with a CP ≥ 0.8 MPa, the internal pores of the soil are closed and the slope of the stress–strain curve does not change significantly.

With the increasing of FTCs, the deformation resistance of the solidified soil decreases. Taking a CP equal to 1.5 MPa as an example, the shear strength at 5, 10, and 14 FTCs for a salinity equal to 1% decreases by approximately 47.3%, 52.6%, and 59.6%, respectively, compared to FTC of 0 times. The shear strengths at a salinity of 2% decrease by 3.6%, 15.5%, and 35.5%, respectively, for increasing FTCs, while those at a salinity of 3% decrease by 8.7%, 23.1%, and 34.6%, respectively, for increasing FTCs. This phenomenon is due to the temperature gradient inside the soil during the FTC. The water moves with the salt to the lower end of the temperature gradient, and the movement of the water and salt damages the internal structure of the solidified soil.

Figure 4 shows that the stress–strain relationship of the saline soil with a salinity equal to 1% indicates either strain hardening or weak strain softening; with increasing salinity the stress–strain curve shows weak strain softening. After the FTCs, the stress–strain relationship of the improve solidified soils showed weak strain hardening and did not change rapidly to strain softening, as noted in the literature [57,58], which indicates that the improvement significantly increased the frost resistance of the saline soils.

### 4.2. Effect of FTCs and Salt Erosion on the Tangent Modulus of Solidified Soils

**Figure 5:**
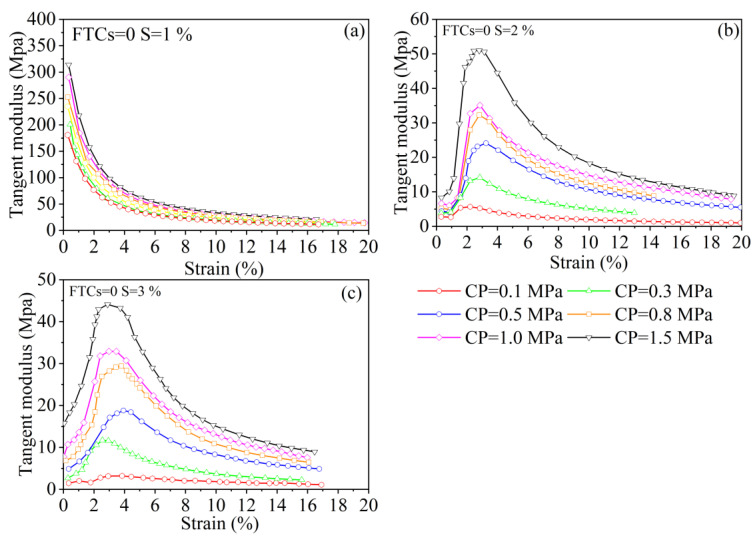
Tangent modulus *E*_s_ for different salinities at different confining pressures. Notes: FTCs represents freeze–thaw cycles, S represents salinity, and CP represents confining pressure.

Figure 5 shows the tangent modulus *E*_s_ for different salinities at different confining pressures; *E*_s_ is an important parameter for measuring a soil’s resistance to deformation. Based on the results of the stress–strain relationship test, the tangent modulus can be calculated for different confining pressures, where the maximum calculated tangent modulus is generally referred to as Young’s modulus. The larger the confining pressure, the larger the tangent modulus. At a salinity of 1%, with an increase in shear stress, the tangent modulus decreases and becomes closer to a certain value. As salinity increases, the tangent modulus shows an increase followed by a decrease, the value of Young’s modulus decreases significantly, and the strain corresponding to the Young’s modulus becomes larger.

**Figure 6:**
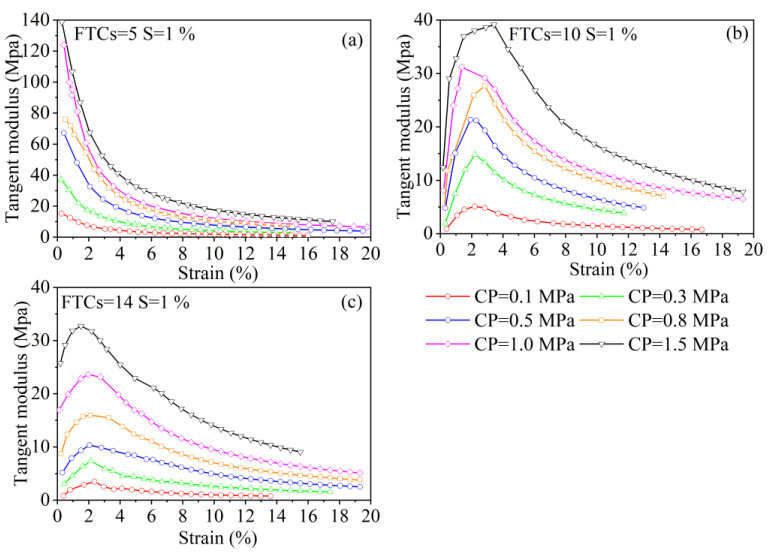
Tangent modulus *E*_f_ for different freeze–thaw cycles with a salinity of 1% at different confining pressures. Notes: FTCs represents freeze–thaw cycles, S represents salinity, and CP represents confining pressure.

Figure 6 shows the tangent modulus *E*_f_ for different FTCs with a salinity of 1% at different confining pressures. At five FTCs, the trend of the tangent modulus resembles that of the unfreeze–thaw; as the shear stress increases, the tangent modulus decreases, but Young’s modulus decreases significantly, and the tangent modulus does not converge to a certain value. As the number of FTCs increases, Young’s modulus continues to decrease and the decreasing trend begins to weaken. The trend of the tangent modulus resembles the trend of the tangent modulus for a salinity greater than 1%, which increases and then decreases. This suggests that FTCs and salinity damage solidified soils in a similar way, with both resulting in a reduction in strength due to additional loosening within the solidified soil.

**Figure 7:**
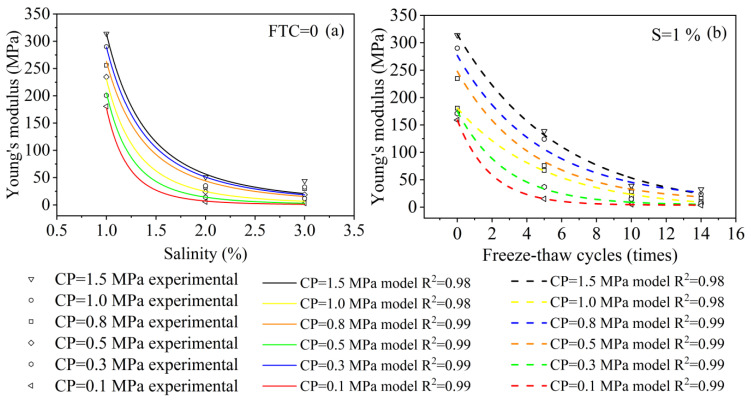
Comparison of test results and calculated results. Notes: FTCs represents freeze–thaw cycles, S represents salinity and CP represents confining pressure.

Figure 7a shows the curves expressing the relationship between Young’s modulus and salinity. The Young’s modulus of the solidified soil shows different rates of decrease with increasing salinity under different confining pressures. Therefore, the Young’s modulus–salinity curve for FTCs = 0 was chosen for analysis, and it was found that the following equation could be used to describe the relationship between *E*_s_ and salinity:

(2) $$E_{S}=a\times x^{b}$$

where *E*_s_ is the Young’s modulus, a and b are calculated parameters, and x is the salinity.

The data obtained from the tests were fitted with the Levenberg–Marquardt optimization algorithm to obtain the values of the calculated parameters a and b for the variation of the Young’s modulus of solidified soil with salinity at different confining pressures, as shown in Table 4. The corresponding correlation coefficients were all greater than 0.977.

To simplify the analysis, using the data in Table 4, the relationship between the parameters a and b and the value of CP was obtained using multiple regression analysis as:

(3) $$a=-346.5x^{2}+1531.05x+1629.17,\;(R^{2}=0.97)$$

(4) $$b=-2x^{2}+4.75x-5.1,\;(R^{2}=0.95)$$

where x is the value of the confining pressure.

Although the R^2^ of the fitting curving are greater than 0.98, it may still be inaccurate due to the three points in salinities. Compared to similar studies [59], the fitting curving demonstrates increasing accuracy with an increase in salinity points. Figure 7a also shows a relative higher inaccuracy with high CP, which may due to the higher CP causing the pore space to reclose. As a result, the Young’s modulus measured at 3% salinity is slightly greater than the predicted value at a high CP. This suggests that the prediction equations provided in this paper should be used to determine the relationship between Young’s modulus and salinity for the solidified salt soils, taking into account whether the CP is too high.

Figure 7b shows the relationship between Young’s modulus and the FTCs; under different CP, Young’s modulus shows different decreasing rates with the increase of FTCs. The error of fitting curving showed a similar regulation in Figure 7a; the reason for this phenomenon is consistent with that previously mentioned. The following equation can be used to describe the relationship between Young’s modulus and FTCs:

(5) $$E_{f}=a+b\times e^{\frac{-x}{c}}$$

where *E*_f_ is the Young’s modulus; *a*, *b*, and *c* are calculated parameters; *e* is a constant; and x is the number of FTCs.

The data obtained from the tests were fitted with the Levenberg–Marquardt optimization algorithm to obtain the values of the calculated parameters *a*, *b*, and *c* for the variation of Young’s modulus of solidified soil with the number of FTCs at different CP, as shown in Table 5. The corresponding correlation coefficients were all greater than 0.977.

Table 5 shows that the parameters *a*, *b*, and *c* all show a linear relationship with CP. To simplify the analysis, the following formula can be used to describe the relationship between parameters *a*, *b*, and *c* and CP:

(6) D=kCP+H

where D represents *a*, *b*, *c*, and *k*; H indicates the calculated parameter values; and CP represents confining pressure. The specific parameter values are shown in Table 6.

### 4.3. Damage Evolution Equations and Intrinsic Structure Modelling

The damage variable under load is defined as *D*_n_, and the damage variable under FTCs is *D*_f_, which means that after N FTCs, the damage to the solidified soil caused by FTCs is characterized by the reduction of the initial elastic modulus:

(7) $$D_{f}=1-\frac{E_{f}}{E_{0}}$$

New damage occurs when the stress level of the soil microelement exceeds a certain strength under load. The damage variable *D*_n_ under load is expressed as the ratio of failure microelement *S*_f_ to total microelement *S* after *N* FTCs:

(8) $$D_{n}=\frac{S_{f}}{S}$$

When the total number of microelements *S* is large enough, Weibull distribution can be used to represent *D*_n_. Therefore, we can allow the probability density function Φ(*F*) of the microelements after experiencing the FTC action to be as follows:

(9) Φ(F;m,F0)=mF0(FF0)em−1xp−FF0m

where *F* is a randomly distributed variable indicating the microelement strength, and the Weibull distribution parameters *m* and *T*_0_ reflect the changes in the average strength and elastic modulus for different FTCs.

Microelement strength is represented by *F*_n_ for a certain range of strengths, i.e., [*F*_n_, *F*_n_ + d*F*_n_], the fracture between the microelements forms damage, and the number of damaged microelements in this process is *S*.

So, Equation (8) can be expressed as:

(10) $$D_{n}=\frac{\int_{-\infty }^{F_{n}}S\Phi (x)dx}{S}=\int_{-\infty }^{F_{n}}\Phi (x)dx$$

Bringing Equation (9) into Equation (10), we obtain:

(11) Dn=1−exp−FnF0m

According to Lemaitre’s stress–strain equivalence hypothesis [60], this is the same as the state of stress under damaged and under virtual non-destructive and effective strain for damaged elastic-brittle materials in the action of real strain ε, namely:

(12) $$\varepsilon =\frac{\delta }{E^{*}}=\frac{\delta^{*}}{E}$$

In which:

(13) $$\delta_{c}A_{c}=\delta_{cd}A_{cd}$$

(14) $$\delta^{*}=\frac{\delta }{1-D}$$

where *E* is the characteristic modulus of nondestructive materials, E^*^ is the characteristic modulus of destructive materials, *δ* is the effective stress acting on the damaged material, δ^*^ is the effective stress acting on the undamaged material, and *D* is the destructive variable. According to the strain equivalence principle extended by Zhang [61], for the undamaged state and the FTC state:

(15) $$\delta_{0}A_{0}=\delta_{f}A_{f}$$

(16) $$D_{f}=\frac{A_{0}-A_{f}}{A_{f}}$$

where *A*_0_ is the initial state of the microelement material-bearing effective area, *A*_f_ is the FTC state of the microelement material-bearing effective area, *δ*_0_ is the effective stress of the initial undamaged microelement material, and *δ*_f_ is the FTC *N* times after the microelement material-bearing effective stress.

Equations (12)–(16) can be combined to obtain:

(17) $$E_{f}=E_{0}(1-D_{f})$$

(18) $$\delta_{f}=E_{0}(1-D_{f})\varepsilon_{f}$$

Equation (18) is the statistical damage model for FTCs if the soil pore space is completely closed due to the confining pressure, which suggests that the soil has reached the nondestructive state, i.e., *D*_f_ = 0.

At this time, the specimen after the FTC is taken as the reference state (undamaged state), and the strain equivalent principle is used again for the specimen under FTC load:

(19) $$\delta_{f}A_{f}=\delta_{n}A_{n}$$

where *A*_n_ is the effective area of the microelement-bearing material in the load state after *N* FTCs and *δ*_n_ is the effective stress of the microelement-bearing material in the load state after *N* FTCs. Therefore, we obtain:

(20) $$\delta_{\mathrm{fd}}=E_{f}(1-D_{n})\varepsilon_{n}$$

where *ε*_n_ is the microelement material strain in the load state after *N* FTCs, and Equation (20) is the statistical damage model under FTC loading.

With the coupling of Equations (17) and (20), we obtain:

(21) $$\delta_{n}=E_{0}(1-D_{\mathrm{al}})\varepsilon_{n}$$

where *D*_al_ =*D*_n_ + *D*_f_ + *D*_f_*D*_n_ indicates the total damage variable of the specimen under load after freeze–thaw cycling.

Substituting Equations (7), (11), and (20) into (21) and considering the influence of the initial damage threshold in the action of the FTCs and loads, the total damage evolution equation becomes:

(22) Dal=1−EfE0,q<q01−EfE0exp−FnF0m,q≥q0

where *q*_0_ is the initial damage stress threshold after *N* FTCs and *q* is the partial stress *q* = *δ*_1_ − *δ*_3_, where *δ*_1_ is the maximum principal stress and *δ*_3_ is the minimum principal stress.

From Equation (22), when the soil pores are completely closed due to the confining pressure, *E*_f_ = *E*_0_, and the total damage evolution equation becomes only the damage equation under load.

In the initial stage of loading, the modified soil partial stress has not yet reached its damage threshold *q*_0_, at which time the deformation of the modified soil microelement obeys Hook’s law, i.e.:

(23) $$\delta_{1}=E\varepsilon_{1}+\upsilon (\delta_{2}+\delta_{3})$$

where *υ* is Poisson’s ratio and *E* is the initial modulus of elasticity of the soil.

Since *δ*_2_ = *δ*_3_ in the conventional triaxial compression test, Equation (23) can be abbreviated as:

(24) $$\delta_{1}=E\varepsilon_{1}+2\upsilon \delta_{3}$$

By combining Equations (14), (22), and (24), considering the influence of the initial damage threshold, the statistical damage equation under freeze–thaw cycle load is:

(25) δ1=Ecε1+2υδ3,q<q0Ecε1exp−FnF0m+2υδ3,q≥q0

As can be seen from Equation (25), to determine the damage variable *D*_al_, it is necessary to solve the microelement strength *F*_n_ at the level of load after characterizing the FTC. Therefore, it is assumed that the micro-failure under the stress level after the FTCs should meet the effective stress and soil material parameters:

(26) Fn−fδ*e=0

where *δ*_e_^*^ is the effective soil micro-cell stress and *f*(*δ*_e_^*^) is the micro-cell stress function. When *f*(*δ*_e_^*^) ≥ *F*_n_, the stress level micro-unit body is damaged after the FTCs. According to the Mohr–Coulomb criterion:

(27) $$\delta_{1}-\delta_{3}\frac{1+\sin\varphi }{1-\sin\varphi }-\frac{2c\cos\varphi }{1-\sin\varphi }=0$$

where *c* and *φ* are the cohesion and the angle of internal friction, respectively. If the microelement strength *F*_n_ damage obeys the Mohr–Coulomb criterion, we have:

(28) $$F_{n}=\delta_{1}^{*}(1-\sin\varphi )-\delta_{3}^{*}(1+\sin\varphi )$$

By combining Equations (28) and (24), an expression for the microelement strength of the soil with respect to the initial confining pressure can be obtained as:

(29) $$F_{n}=(E\varepsilon_{1}+2\upsilon \delta_{3})(1-\sin\varphi )-\delta_{3}^{*}(1+\sin\varphi )$$

The damage statistical model developed in this paper uses three-parameter Weibull distribution to describe the damage evolution equation for solidified soils, where the microelement strength *F*_n_ is determined by the strength criterion for geotechnical soils, i.e., Equation (29). Therefore, only the parameters *m* and *F*_0_ need to be determined; these can be calculated from the characteristic points of the modified soil stress–strain curve. At different confining pressures, the solidified soil reaches its peak strength with a stress increment of 0, i.e., with:

(30) $$\frac{\partial \delta_{1}}{\partial \varepsilon_{1}}\left|{}_{\begin{array}{c} \delta_{1}=\delta_{m}\\ \varepsilon_{1}=\varepsilon_{m} \end{array}}\right.=0$$

where *δ_m_* is the stress corresponding to the peak stress–strain curve of solidified soil and *ε_m_* is the strain corresponding to the peak stress–strain curve of the solidified soil.

Differentiating Equation (25) and writing it according to Equation (22) can be simplified to:

(31) $$m\left[-{\left(\frac{F_{n}}{F_{0}}\right)}^{m-1}\right]=\frac{F_{0}}{\left(1-\sin\varphi \right)}$$

When the solidified soil reaches its peak strength, its stress point should satisfy the following expression:

(32) $$\delta_{m}=E_{0}(1-D_{al})\varepsilon_{m}$$

Combining Equations (31) and (32), we obtain:

(33) $$m=\frac{(E_{0}\varepsilon_{m}+2\upsilon \delta_{3})\left(1-\sin\varphi \right)-\delta_{3}\left(1+\sin\varphi \right)}{\left(1-\sin\varphi \right)\mathrm{In}\left(\frac{\delta_{m}}{\varepsilon_{m}E_{f}}\right)}$$

(34) F0=(E0εm+2υδ3)1−sinφ−δ31+sinφInεmEfδm1m

**Figure 8:**
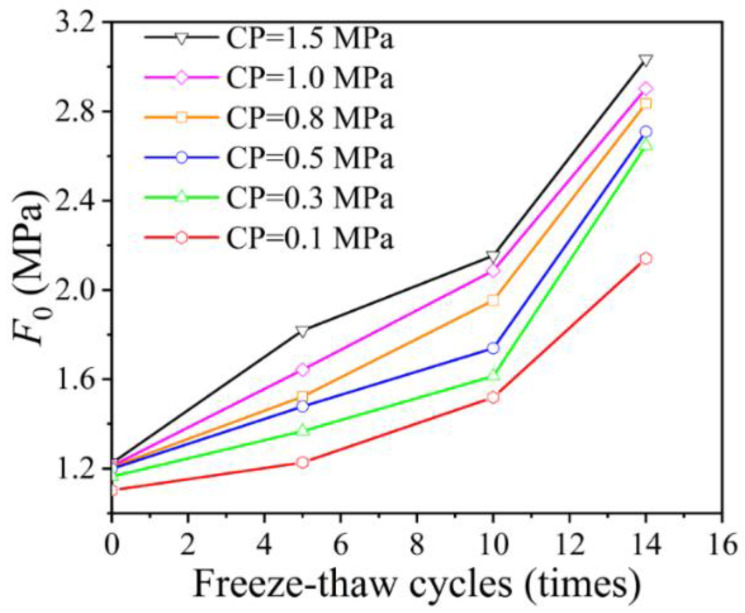
Effect of freeze–thaw cycles and confining pressure on *F*_0_. Notes: CP represents confining pressure.

It can be seen from Equation (33) that parameter m is positively correlated with *F*_0_, and the influence of FTCs on the parameters is similar. Therefore, *F*_0_ is taken as an example to show the influence of FTCs and CP on the parameters. As shown in Figure 8, the value of parameter *F*_0_ without FTCs varies from 1.1 to 1.22. The greater the confining pressure, the greater the *F*_0_. With an increase in FTCs, the growth rate of *F*_0_ is related to the confining pressure. After five FTCs, the *F*_0_ of CP = 1.5 increases by 48.4%, and the *F*_0_ of CP = 0.1 only increases by 11.3%. After 14 cycles, the *F*_0_ of CP = 1.5 increases by 147.6%, and the *F*_0_ of CP = 0.1 increases by 94.2%.

**Figure 9:**
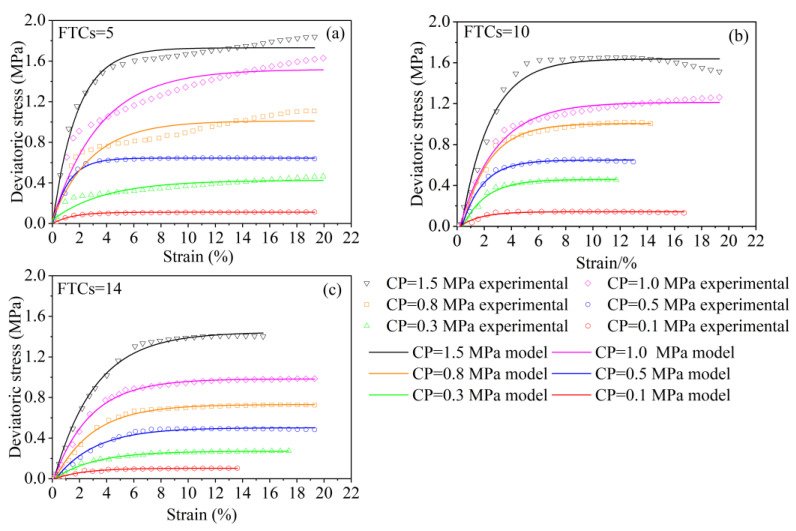
Theoretical stress–strain curves for calculated and tested values of solidified soils.

Now, bringing the model parameters into Equation (25), the solidified soil theoretical stress–strain curve considering the damage threshold value under FTC and load can be obtained and compared with the experimental curve, as shown in Figure 9. As seen in the figure, when the number of FTCs equals five, the total stress–strain curve is significantly lower than that of the samples with more than five FTCs, and the shear stress begins to decrease at this time. With increasing CP, the slope of the linear phase change curve increases significantly, and the threshold value of initial damage stress increases gradually. The lower the CP, the higher the visibility, which shows that high CP has the effect of reducing the initial damage caused by FTCs.

The experimental results are in good agreement with the theoretical calculation results. The theoretical results reflect the overall trend of the strength and deformation of the solidified saline soil under FTCs and loads with the number of FTCs and CP, indicating that the proposed freezing–thawing damage model of solidified soil can relatively reflect the entire stress–strain process in a temperature-alternating environment, and under external load, even though it could be inaccurate under a high confining pressure inducing the effect of pore closure.

## 5. Conclusions

In this paper, the UCST of solidified saline soil was conducted in the laboratory at various salinities, temperatures, and ages, in order to analyze the coupling effect of freezing and salt. Young’s modulus was investigated by CU triaxial tests, and a damage constitutive model based on Weibull distribution is proposed for solidified saline soil under the effect of FTCs. The main conclusions are as follows:

1. After using lime, fly ash, and cement to cure saline soils in cold regions, the UCS is greatly improved, and the solidified saline soils with a salinity of less than 3% can meet the requirements of the sub-base filler for secondary and lower roads with medium and light traffic at the age of 14 d. Both frost heave and salt erosion could cause irreversible damage in soil, and the coupling effect of freezing-salt will amplify the rate of strength loss.
2. After curing the saline soils in cold regions, the freeze–thaw resistance of the solidified saline soils is remarkably enhanced, and the stress–strain curve of the solidified saline soils does not show rapid strain softening after FTCs. The effect of FTCs and salt erosion causes peak stress to shift backward at the appearance of Young’s modulus, which indicates that, with increased FTCs and salinity, the resistance to deformation of the solidified saline soil was reduced.
3. Considering the damage threshold of FTCs on solidified saline soil, Weibull distribution is used to describe its damage evolution regulation. The simulation results of modelling show that the damage constitutive model is more suitable than the traditional constitutive model for solidified saline soil under the effect of FTCs.

## Figures and Tables

**Table 1:** Average values of basic physical and mechanical properties of soils.

Specific Gravity (g/cm^3^)	Optimum Moisture Content (%)	Dry Density (g/cm^3^)	Liquid Limit (%)	Plastic Limit (%)	Plastic Limit Index	Uniformity Coefficient	Coefficient of Curvature
1.8	16.6	1.7	33	21	12	6.04	1.4

**Table 2:** Chemical composition of inorganic improvers.

Chemical Composition	SiO_2_	CaO	MgO	SO_3_	Loss on Burn	Al_2_O_3_	Fe_2_O_3_
Lime	3.5	82.4	7.3	0.6	2.0	/	/
Fly ash	51	3.87	0.93	0.60	1.44	32.30	7.56
Cement	21	59	≤1.5	≤0.3	≤0.5	15.1	4.2

**Table 3:** Factors and levels.

	Factor	Levels
Stage 1	Temperature (°C)	20/−10/−20
	Age (d)	3/7/14/28/90
	Salinity (%)	0/1/2/3/4/5
Stage 2	Freeze–thaw cycles (times)	0/5/10/14
	Salinity (%)	1/2/3
	Confining pressure (MPa)	0.1/0.3/0.5/0.8/1.0/1.5

**Table 4:** Calculation of parameter a and b values.

CP/Mpa	a	b	*R* ^2^
0.1	1806.7	−4.9	1.00
0.3	2007.3	−3.6	0.99
0.5	2344.1	−3.0	0.99
0.8	2552.2	−2.6	0.98
1.0	2895.5	−2.5	0.98
1.5	3130.4	−2.3	0.94

**Table 5:** Calculated values for parameters a, b, and c.

CP (MPa)	*a*	*b*	*c*	*R* ^2^
0.1	26.5	1552.1	1.9	1.00
0.3	44.2	1635.4	3.0	0.99
0.5	54.7	1834.9	3.7	0.98
0.8	90.6	2268.4	4.2	1.00
1.0	117.7	2964.4	5.2	0.98
1.5	202.2	3203.9	5.9	0.98

**Table 6:** Calculated parameter values *k*, H.

Parameter	*k*	H	*R* ^2^
*a*	124.3	2.3	0.96
*b*	1328.3	1313.3	0.93
*c*	2.8	2.0	0.94

## Data Availability

The data used to support the findings of this study are included within the article.
